# Smartphone Use During School Hours and Association With Cognitive Control in Youths Aged 11 to 18 Years

**DOI:** 10.1001/jamanetworkopen.2026.1092

**Published:** 2026-03-09

**Authors:** Eva H. Telzer, Kaitlyn Burnell

**Affiliations:** 1 Department of Psychology and Neuroscience, University of North Carolina, Chapel Hill

## Abstract

**Importance:**

Smartphones are a pervasive feature of adolescents’ daily lives, raising concern about how smartphones are used in contexts such as school that require sustained attention and self-regulation.

**Objectives:**

To describe youths’ smartphone use during each hour of the school day and examine whether smartphone use during school is associated with poorer cognitive control, a key developmental process underlying academic success.

**Design, Setting, and Participants:**

This cross-sectional study of youths aged 11 to 18 years from the Southeastern US objectively assessed smartphone use every hour for 14 consecutive days between April 8, 2021, and February 2, 2022 (cohort 1), and February 1, 2023, and December 11, 2024 (cohort 2), providing thousands of data points to capture actual engagement.

**Main Outcomes and Measures:**

The iPhone iOS (Apple) screen time report captured smartphone use at every hour. Cognitive control was measured in the older cohort using a go/no-go task, with the signal detection metric *d*ʹ quantifying inhibitory control.

**Results:**

A total of 79 participants (mean [SD] age, 15.10 [2.04] years; 41 [51.9%] female) participated in the study. Youths were using their smartphones during every hour of the school day, spending a total of 2.22 hours of the school day on their smartphones. Youths aged 15 to 18 years spent more time on their smartphones during school hours than those aged 11 to 14 years (mean [SD], 23.28 [18.34] vs 11.57 [16.83] min/h; *F*_1,76_ = 28.82, *P* < .001, η^2^ = 0.28). Youths spent a mean (SD) of 40.14 (39.56) minutes on social media and 13.85 (25.22) minutes on entertainment apps during school hours. Youths checked their smartphones a mean (SD) of 64.46 (32.83) times during school hours. More frequent smartphone checking was associated with lower *dʹ* values (*F*_1,28_ = 4.8, *P* = .04, η^2^ = 0.15), indicating poorer cognitive control.

**Conclusions and Relevance:**

This cross-sectional study found that youths use smartphones approximately one-third of the school day; this use was associated with reduced cognitive control. These findings highlight the need for school-level policies and digital literacy programs that address not only overall screen time but also habitual smartphone-checking behaviors that fragment attention.

## Key Points

**Questions:**

How much are youths using their smartphones during school instruction time, and what is the association of this use with cognitive control?

**Findings:**

This cross-sectional study of 79 youths aged 11 to 18 years found that they used their smartphones during every hour of the school day, spending one-third of their school day on their smartphones. This use was associated with reduced cognitive control.

**Meaning:**

This study’s results suggest that frequent smartphone use during school reflects broader challenges in self-regulation with implications for learning and cognitive development.

## Teaser

This cross-sectional study of adolescents examines whether smartphone use during the school day is associated with poorer cognitive control.

## Introduction

Smartphones are a pervasive feature of adolescents’ daily lives, with more than 95% of US teens reporting access to a smartphone and nearly half describing themselves as “almost constantly” online as of 2024.^1^ This ubiquity has raised concern about how smartphones shape adolescents’ development, particularly in contexts such as school that are designed to foster sustained attention, academic engagement, and social growth.

With smartphones providing constant mobile access, social media distraction—when social media diverts attention from an ongoing task^2,3^—readily permeates classrooms. Developmental theories of self-regulation suggest that adolescence is a period of heightened vulnerability to distraction, given ongoing maturation of prefrontal cognitive control systems alongside sensitivity to rewarding social information.^4^ The constant availability of smartphones therefore will increase social media distraction during school hours, creating unique challenges for adolescents’ ability to regulate attention and maintain focus on academic tasks.

Two recent studies^5,6^ used passive sensing methods to show that adolescents spend substantial time on their smartphones during school, primarily on social media. However, these studies aggregated use across the full school day, making it unclear whether smartphone use occurs mainly during breaks (eg, lunch) or intrudes on classroom time. These are the only studies, to our knowledge, that have objectively looked at smartphone use during school among adolescents; there remains a significant gap in the literature in understanding the temporal dynamics of when adolescents are most likely to use their devices during school and what this means for adolescents’ cognition.

The current study builds on this emerging literature. First, we provide a fine-grained description of youths’ smartphone use during school by examining screen time at each hour of the school day, advancing prior work that aggregated use across the school day.^5,6^ We also test differences between youths in high school (aged 15-18 years) and middle school (aged 11-14 years), hypothesizing that high school students would engage in greater screen time during school hours, consistent with prior research.^5^ Youths’ difficulty disengaging from their smartphones during school likely stems from self-control failure—the inability to resist the short-term gratification of checking social media in favor of long-term goals, such as academic achievement.^2,7^ Theoretical frameworks suggest that smartphone use may undermine attention through multiple pathways. The limited capacity model of mediated message processing^8^ posits that frequent smartphone use depletes attentional resources, whereas the interference hypothesis^9^ suggests that time and attention spent on smartphones come at the expense of activities essential for learning and social development.

Second, we tested whether smartphone use during school is associated with cognitive control, a key developmental process underlying academic success and self-regulation. We examined both overall screen time and habitual smartphone use (eg, frequent checking), the latter of which has been associated with longitudinal differences in prefrontal cortex activation, which subserves cognitive control.^10^ We hypothesized that more frequent smartphone checking (but not overall screen time) would be associated with poorer cognitive control, reflecting the depletion of attentional resources.

## Methods

### Participants

Participants included 79 youths aged 11 to 18 years recruited from the Southeastern US (Table 1). The sample size was based on attaining as many participants as possible within the data collection phase. An a priori power analysis was not calculated. The older cohort included participants in high school (aged 15-18 years) recruited from a larger longitudinal study examining adolescent digital technology use. The analytic sample was part of wave 5 of the study, which took place from April 8, 2021, to February 2, 2022. Wave 5 initially included 95 youths. Sixteen were excluded because they had Android devices (which did not provide the granular hourly data of focus in the current research); an additional 8 were excluded because they had iOS devices but did not provide usable data. Twenty were excluded because they did not have smartphone data during school hours (ie, participation occurred during the summer months). The final analytic sample of 51 youths did not differ from the excluded sample of 44 youths on age, income, race and ethnicity, gender, or cognitive control. The younger cohort included 28 youths in middle school (aged 11-14 years) recruited from the same Southeastern US county, as part of wave 1 of a separate longitudinal study, which took place from February 1, 2023, to December 11, 2024. The larger sample initially included 92 youths. Twenty were excluded because they did not have a smartphone, and 20 additional adolescents were excluded because they had Android devices. Of the remaining 52 smartphone users, 22 were excluded because they did not have smartphone data during school hours, and 2 were excluded because they did not upload usable data. The final analytic sample of 28 youths did not differ from the excluded sample of 64 youths on age, income, race and ethnicity, or gender. All participants came from the same school district. Participants completed written assent and parents completed written consent in accordance with the University of North Carolina at Chapel Hill Institutional Review Board. Participants were compensated up to $50 for providing screenshots of their screen use each day for 14 days. The current study followed the Strengthening the Reporting of Observational Studies in Epidemiology (STROBE) reporting guideline for cross-sectional studies.

**Table 1.** Characteristics of the Study Sample^a^

Characteristic	No. (%) of participants^b^	
	Older cohort (aged 15-18 years) (n = 51)	Younger cohort (aged 11-14 years) (n = 28)
Age, mean (SD), y	16.5 (0.6)	13.0 (0.8)
Family income, mean (SD), $	45 000-59 999	50 000-60 000
Gender		
Male	22 (43.1)	15 (53.6)
Female	28 (54.9)	13 (46.4)
Nonbinary	1 (2)	0
Race and ethnicity		
Asian	2 (3.9)	0
Black	13 (25.5)	6 (21.4)
Latine or Hispanic	18 (35.3)	10 (35.7)
Native Hawaiian	0	1 (3.4)
White	15 (29.4)	5 (17.2)
Multiracial	3 (5.9)	2 (6.9)

^a^ Youths self-reported their gender identity and race by selecting predefined categories. Family income was reported by the primary caregiver based on selecting predefined categories. The income categories varied slightly in the younger and older cohorts. The older cohort had available data on screen time, total pickups, and cognitive control task. The younger cohort had available data on screen time, social medical time, and entertainment time.

### Smartphone Use

Each day, youths were sent a daily questionnaire to complete via ExpiWell,^11^ an application that they downloaded to their personal smartphone. They completed a self-report measure indicating whether it was a school day. Participants in both cohorts were asked to upload 3 screenshots to the ExpiWell app on their smartphone each day of their smartphone’s screen time report from the prior day, which showed a graphical display of their smartphone use per hour for the 24-hour day (eFigure in Supplement 1). From these graphical displays, we used a raster graphic editor to decode the exact value of smartphone use per hour.

To maximize the information we could collect and minimize participant burden, we obtained different metrics for each cohort. Thus, we collected 3 screenshots for the younger cohort (overall screen time, social media, and entertainment) and 3 screenshots for the older cohort (overall screen time, pickups, and notification, which was not included in the current analyses). Had we asked both cohorts to do everything (ie, 6 screenshots per day), we risked lower adherence. For the older youth cohort, daily screenshots were uploaded to capture (1) overall screen time, (2) total pickups, and (3) total notifications (which were not used in the current study). For the younger youth cohort, screenshots were uploaded to capture (1) overall screen time, (2) social networking screen time (eg, Facebook, Instagram, TikTok, and Snapchat), and (3) entertainment screen time (eg, YouTube).

Because all participants came from the same school district, we knew the start and end times of their school each day (8 am-3 pm) and thus could examine smartphone use restricted to school hours. All school days were fully in person for both cohorts of adolescents.

Across the 2-week data collection period, there were a maximum of 10 school days (and 4 weekend days) per participant and screen use at every hour of the day (ie, 24 data points per day). Thus, we obtained up to 336 data points per participant, with up to 70 data points per participant for screen time during school hours. Not all participants uploaded screen time reports every day. Participants uploaded a mean (SD) of 6.03 (2.94) days of screen time data, with most participants submitting 9 days. Participants provided a mean (SD) of 42.18 (20.58) hours (ie, data points) during school time, with a range of 7 to 70 data points during school. We had a total of 16 547 data points (including nonschool days and nonschool hours) for screen time across the 14 days (11 568 for the older cohort and 4979 for the younger cohort). There were 10 967 total data points for screen time during school days (7356 data points for the older cohort and 3611 for the younger cohort) and 3498 data points for screen time during school hours (2114 data points for the older cohort and 1057 data points for the younger cohort). In addition, there were 2107 data points for pickups during school hours, 980 data points for social networking screen time during school hours, and 854 data points for entertainment screen time during school hours.

### Inhibitory Control Task (Planets Task)

The older cohort of youths were invited to an in-person laboratory visit and completed an incentive-boosted inhibitory control task, which has been used in prior research^12^ to reliably measure cognitive control in adolescents. Participants were presented with an image of a planet on the screen and were instructed to press a button whenever they saw a crater planet (ie, go trials) but withhold their response (ie, not press the button) whenever they saw a striped planet (ie, no-go trials). If participants successfully pressed a button after seeing a crater planet or avoided pressing a button after seeing a stripped planet, then they earned a monetary reward but lost a monetary reward if they made an incorrect response. Participants were paid a monetary reward based on their earnings. Participants saw a cue at the beginning of each round that indicated how much they could earn. A pink cue indicated they could earn $1 for correct responses and lose $0.50 for incorrect responses on each trial (ie, big boost), a blue cue indicated they could earn $0.20 for correct responses and lose $0.10 for incorrect responses on each trial (ie, small boost), and a yellow cue indicated they would not win or lose money (ie, no boost). The signal detection theory metric dʹ is commonly used in go/no-go tasks to quantify cognitive control.^13^ It measures the ability to discriminate between signals.^14^

### Statistical Analysis 

We extracted smartphone data for each hour of the school day. We ran analyses to calculate descriptives (eg, mean [SD] and range) for screen time at each hour of the school day. We conducted analyses of variance (ANOVA) to test for differences between the younger and older cohorts and examined smartphone use during school hours vs after school and on school days vs nonschool days. Lastly, we conducted ANOVAs to examine associations between frequency of smartphone checking and *d*ʹ on the cognitive control task. Analyses were run in SPSS, version 31.0 (SPSS Inc). A 2-sided *P* < .05 was considered statistically significant.

## Results

### Screen Time During School

Across the full sample of 79 participants (mean [SD] age, 15.10 [2.04] years; 41 [51.9%] female, 37 [46.8%] male, and 1 [1.3%] nonbinary; 2 [2.5%] Asian, 19 [24.1%] Black, 28 [35.4%] Latine or Hispanic, 1 [1.3%] Native Hawaiian, 20 [25.3%] White, 5 [6.3%] multiracial, and 4 [5.1%] missing race data)(Table 1), we computed screen time across the 14-day period for each hour of the day as well as summed across the school day (Table 2). Youths were on their smartphones during every hour of the school day, ranging from a mean (SD) of 16.01 (17.98) minutes at 8 am to 22.26 (18.43) minutes at 2 pm. Youths spent about a total of 2.22 hours of the school day on their smartphones, accounting for 28.5% of their mean 24-hour phone use of 7.78 (3.76) hours daily. This ranged from a minimum of 8 minutes on their smartphones during school to a maximum of 5.33 hours. There was not a single participant in the sample who did not use their smartphone during school.

**Table 2.** Hourly and Daily Mean of Screen Time, Social Media, Entertainment, and Pickups During School Hours

Variable	Median (range) [IQR]	Mean (SD)	Skew
Hourly screen time (n = 79 participants and 453 data points)			
8 am	10 (0-60) [1-26]	16.01 (17.79)	1.09
9 am	11 (0-60) [1-33]	18.48 (19.64)	0.79
10 am	12 (0-60) [2-34]	19.28 (19.46)	0.77
11 am	15 (0-60) [5-32]	19.21 (17.44)	0.79
12 pm	15 (0-60) [2-36]	20.13 (19.3)	0.64
1 pm	16 (0-60) [5-33]	20.28 (18.15)	0.71
2 pm	17 (0-60) [6-36]	22.26 (18.43)	0.6
Hourly social media time (n = 28 participants and 140 data points)			
8 am	0 (0-60) [0-4]	4.28 (9.51)	3.18
9 am	0 (0-60) [0-3]	4.58 (11.19)	3.01
10 am	0 (0-52) [0-5]	4.36 (8.68)	2.54
11 am	1 (0-44) [0-7]	5.04 (8.65)	2.47
12 pm	0 (0-58) [0-7]	5.66 (11.44)	2.71
1 pm	1 (0-60) [0-5]	5.87 (11.75)	2.38
2 pm	3 (0-57) [0-13]	8.39 (11.91)	1.68
Hourly entertainment time (n = 28 participants and 122 data points)			
8 am	0 (0-46) [0-0]	1.19 (5.27)	5.79
9 am	0 (0-47) [0-0]	1.44 (6.22)	5.13
10 am	0 (0-40) [0-0]	2.17 (8.74)	4.04
11 am	0 (0-55) [0-0]	3.65 (9.25)	3.25
12 pm	0 (0-59) [0-0]	3.2 (10.18)	3.84
1 pm	0 (0-60) [0-0]	3.28 (9.73)	4.24
2 pm	0 (0-58) [0-1]	3.48 (10.52)	3.76
Hourly pickups (n = 51 participants and 301 data points)			
8 am	6 (0-54) [2-11]	7.83 (7.26)	1.86
9 am	6 (0-41) [3-12.5]	8.18 (7.39)	1.36
10 am	7 (0-33) [3-12]	8.74 (7.14)	1.09
11 am	9 (0-41) [5-14]	10.08 (7.65)	1.13
12 pm	8 (0-46) [3-14]	9.52 (8.24)	1.39
1 pm	8 (0-42) [5-14]	9.91 (7.52)	1.26
2 pm	8 (0-41) [4-15]	10.2 (8.21)	1.02
Total screen time (8 am-2:59 pm) (n = 79 participants and 3498 data points)	109 (8-319.67) [82.88-182.9]	133.18 (79.5)	0.51
Total social medial time (8 am-2:59 pm) (n = 28 participants and 980 data points)	32.33 (0-177.57) [10.22-59.75]	40.14 (39.56)	1.98
Total entertainment times (8 am-2:59 pm) (n = 28 participants and 854 data points)	4.42 (0-107.1) [0.41-13.2]	13.85 (25.22)	2.92
Pickups (8 am-2:59 pm) (n = 51 participants and 2107 data points)	59.88 (13.25-143.5) [37.5-93.33]	64.46 (32.83)	0.41

#### Age Differences in Screen Time During School

**Figure 1.**
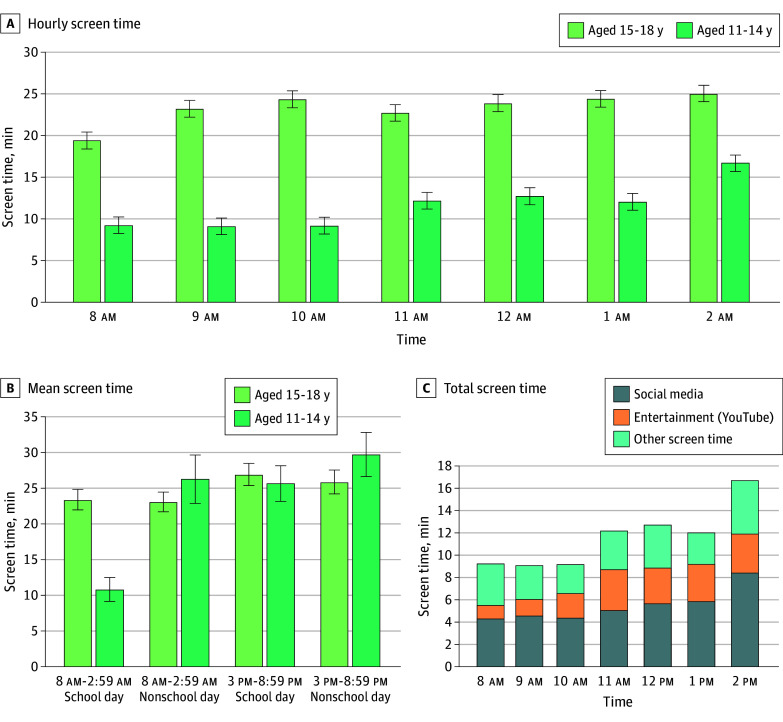
Bar Graphs of Smartphone Screen Time in the Younger and Older Cohorts A, Hourly screen time among the younger and older cohorts across the school day. Screen time was significantly higher across the school day in the older cohort. Screen time increased across the school day (eg, from 8 am to 12 pm [*t*_77_ = 2.19, *P* = .03]; from 10 am to 2 pm [t_77_ = 2.13, *P* = .03]). B, Mean screen time in the younger and older cohorts on school days and nonschool days. C, Total screen time on social media, entertainment, and other screen time at each hour of the school day in the younger cohort. Error bars indicate SDs.

To test for differences in screen time by age and time of day, we conducted a 2- (younger and older cohorts) × 7-way (8 am, 9 am, 10 am, 11 am, 12 pm, 1 pm, and 2 pm) ANOVA. We found a significant between-person effect such that the older cohort (mean [SD], 23.28 [18.34] min/h) spent more time on their smartphones during school hours than the younger cohort (mean [SD], 11.57 [16.83] min/h; *F*_1,76_ = 28.82, *P* < .001, η^2^ = 0.28). We also found a within-person effect of hour such that youths tended to increase their screen time during the school day (*F*_6,456_ = 3.76, *P* < .001, η^2^ = 0.05) (Figure 1A). There was no interaction between cohort and hour (*F*_6,456_ = 0.91, *P* = .49).

Next, we computed mean time spent on the smartphone across the 14 days during school hours (8 am-2:59 pm) and after school hours (3 pm-8:59 pm). We computed these separately for school days and nonschool days. We conducted a 2 (younger and older cohorts) × 2 (school hours and after school hours) × 2-way (no school vs school day) ANOVA to test for screen time differences by age, time of day, and school day. We found a significant 3-way interaction (*F*_1,68_ = 6.72, *P* = .01, η^2^ = 0.09). As shown in Figure 1B, younger youths logged less screen time than older youths but only during school hours on school days (*t*_76_ = 5.37, *P* < .001). They did not differ during school hours on nonschool days (*t*_68_ = 0.38, *P* = .76) or after school on school days (*t*_76_ = 0.44, *P* = .66) or nonschool days (*t*_68_ = 0.85, *P* = .40). Within the younger cohort, they reduced their screen time during school hours on school days relative to nonschool days (*t*_20_ = 4.02, *P* < .001).

#### Social Media and Entertainment Screen Time During School Hours

In the younger cohort, we examined the distribution of screen time across social media, entertainment, and other screen time for each hour of the school day (Table 2). Youths spent a mean (SD) of 40.14 (39.56) minutes total on social media. This ranged from a minimum of 0 minutes on social media during school to a maximum of approximately 3 hours. There was only 1 participant in the sample who did not use social media on their smartphone during school. For entertainment, youths spent a mean (SD) of 13.85 (25.22) minutes total during school hours. This ranged from a minimum of 0 minutes during school to a maximum of 1.76 hours. Only 3 participants in the sample did not use entertainment on their smartphones during school. Youths used social media and entertainment during every hour of the school day (Figure 1C). Social media and entertainment accounted for 69.8% of their total screen time during school hours.

### Smartphone Use and Cognitive Control

In the older cohort we examined the frequency of smartphone pickups for each hour of the school day (Table 2). Youths were checking their phones during every hour of the school day, ranging from a mean (SD) of 7.83 (7.26) times at 8 am to 10.20 (8.21) times at 2 pm. Youths checked their smartphones a mean (SD) of 64.46 (32.83) times during school, ranging from 13.25 to 143.50 times. There was not a single participant in the sample who did not check their smartphone during school.

**Figure 2.**
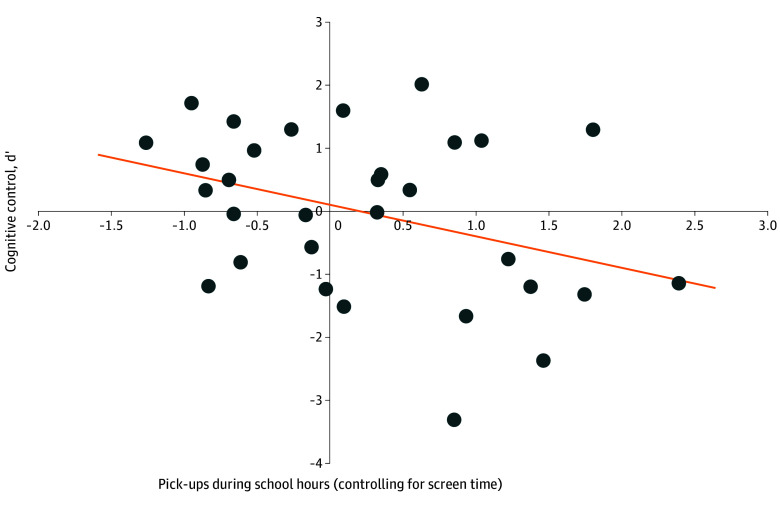
Scatterplot of the Association of Mean Pickups per Hour During School With Cognitive Control Pickups were controlled for screen time. Solid line indicates the regression.

Next, we computed a repeated-measures ANOVA with incentive (big boost, small boost, or no boost) as a within-subject factor and pickups and screen time during school hours as between-person factors, controlling for the number of days participants uploaded screen time reports. We did not find a main effect of incentive on cognitive control (*F*_2,56_ = 0.10, *P* = .91) or an interaction between incentive and total pickups (*F*_2,56_ = 1.07, *P* = .35) or screen time (*F*_2,56_ = 1.51, *P* = .23). We found a significant association of pickups during school with mean *d*ʹ (*F*_1,27_ = 4.87, *P* = .04, η^2^ = 0.15) but no association with screen time (*F*_1,27_ = 0.17, *P* = .68). As shown in Figure 2, youths who picked up their smartphones more during school (controlling for screen time during school) exhibited lower *d*ʹ, indicating impaired cognitive control.

## Discussion

Smartphones have become a defining feature of adolescent daily life, raising increasing concern about how constant connectivity may shape attention, learning, and self-regulation. Although much research has documented adolescents’ overall screen time, less is known about how smartphones are used throughout the school day and how such patterns are associated with cognitive control. By assessing smartphone use objectively at every hour for 2 weeks, yielding thousands of data points, the current study provides one of the most detailed, naturalistic accounts of smartphone use during school among youths aged 11 to 18 years.

Our results show that adolescents use their smartphones at every hour of the school day. On average, they spent more than 2 hours on their smartphones during school—nearly one-third of their total daily use. No participants abstained from smartphone use during school, and 1 in 4 spent more than 3 hours on their smartphones during school. These findings extend those of recent studies^5,6^ that aggregated smartphone use across the school day. By analyzing smartphone use hourly, our data reveal that engagement is not confined to breaks, such as lunch, but is ubiquitous across every hour of the school day. This pattern suggests that smartphone use is a continuous and pervasive source of distraction, underscoring how embedded smartphones have become in adolescents’ daily lives, even in structured academic contexts.

Although smartphone use was ubiquitous, we observed age-related differences. Youths aged 15 to 18 years spent more time on their smartphones during school than youths age 11 to 14 years. The younger cohort reduced their smartphone use during school on school days compared with nonschool days, whereas the older cohort did not. Both groups showed similar levels of smartphone use after school, suggesting that younger adolescents may still respond to school-related structure or monitoring, whereas such constraints diminish with age. These findings highlight developmental changes in social motivation and self-regulation that interact with the increasing autonomy afforded by smartphones. Early adolescence may represent a window of opportunity for promoting healthier digital habits because younger adolescents may be more responsive to contextual constraints. It is also possible that increasing smartphone restrictions in schools explains this pattern because the younger cohort participated 2 years after the older cohort. The older cohort also participated closer to the COVID-19 pandemic (2020-2021), although all adolescents were back in school and no remote learning was occurring during data collection. Future research should examine how school environments, parental involvement, and monitoring influence these patterns.

A closer look at the younger cohort showed that social media and entertainment accounted for more than 70% of screen time during school hours, consistent with prior research^5,6^ showing that most smartphone use during school is used for social media. The prevalence of social media use throughout the school day may reflect both the high salience of online social interactions and the difficulty of disengaging from digital content. Unlike the delayed rewards of academic achievement, social media provides immediate and variable reinforcement, known to drive compulsive behaviors.^15^ As a result, adolescents may become conditioned to seek constant stimulation and struggle with the sustained attention required for deep learning.

To test associations between smartphone use and self-regulation, we examined whether smartphone use was associated with cognitive control, measured with a go/no-go task. The finding that frequency of phone pickups—but not total screen time—was associated with poorer cognitive control is consistent with theories of attentional fragmentation and cognitive load. Repeated pickups create recurrent task switching, which disrupts sustained attention and depletes limited executive resources. Within the limited capacity model of mediated message processing, frequent interruptions draw on finite cognitive resources needed to maintain goal-directed behavior.^8^ In parallel, the interference hypothesis suggests that frequent pickups repeatedly redirect attention away from activities essential for learning and social development.^9^ Frequent, brief interruptions likely fragment attention and diminish sustained focus, consistent with prior work showing that digital interruptions can impair learning and academic performance^16^ and increasing social media use is linked to later poorer cognitive performance.^17^ Related work on task switching and media multitasking further demonstrates costs to executive control from frequent attentional shifts.^18^ Our findings also align with evidence that individuals who engage in problematic social media use perform worse on inhibitory control tasks.^19^ Together, these findings suggest that frequent smartphone checking during school reflects broader challenges in self-regulation. It is also possible that adolescents with more difficulty controlling their attention may be more inclined to check their smartphones during school. Either way, these findings suggest that failures in cognitive control develop in tandem with frequent smartphone checking during school hours. Although we did not find that total screen time during school was associated with poorer cognitive control, screen time may be detrimental to other aspects of cognition, such as learning and achievement, because prior research has shown that greater screen time is associated with lower academic achievement^20^ and impacts the learning of peers nearby.^21^

### Limitations

This study has limitations. Although the sample included thousands of data points among an ethnically and socioeconomically diverse sample, the results of this study should be interpreted with some caution given the relatively small sample size, that data collection occurred during 2 different sampling periods for the younger and older cohorts, and that Android users were excluded. Moreover, we relied on the App Store’s built-in categorization for social networking vs entertainment applications, which places some social media apps into entertainment (eg, YouTube) and may not accurately capture all social media platforms,^22^ and therefore the values for social media may be higher. Although social networking and entertainment categories were by far the most used categories, the small amount of other screen time may be used for a variety of tasks, such as calculators, calendars, cameras, and music. Future research should capture specific application use throughout school. Although we were able to capture the full school day at the hourly level, we are unable to formally differentiate instructional from informal times during school hours (eg, passing periods and lunch time). Lastly, the reason for frequent smartphone pickups during school are unknown (eg, checking the time vs checking social media) and should be further unpacked in future research. These data were collected before state-level smartphone bans in schools; therefore, findings may vary across regions as device restrictions in schools increase.

## Conclusions

This cross-sectional study of adolescents provided a detailed analysis of smartphone use during the school day—one in which smartphones are omnipresent, heavily used for social and entertainment purposes, and associated with poorer cognitive control. Notably, smartphone bans are not sufficient for addressing the pervasive and often detrimental impact of smartphones on adolescents’ cognitive and social well-being.^23,24^ That adolescents used their smartphones across every hour of the school day underscores the need for school-level policies and digital media literacy initiatives that directly address the impact of smartphone use and social media on adolescents’ cognitive and social well-being.

## Notes

Supplement 1. **eFigure.** Screen Shot

Supplement 2.
Data Sharing Statement
