# Supplementary material for: Smartphone Use During School Hours and Association With Cognitive Control in Youths Aged 11 to 18 Years
Source: JAMA Netw Open. 2026 Mar 9;9(3):e261092. doi: 10.1001/jamanetworkopen.2026.1092 (PMC12973097; doi:10.1001/jamanetworkopen.2026.1092)
Supplement: Supplement 1. — eFigure. Screen Shot [file jamanetwopen-e261092-s001.pdf]

Supplemental Online Content

Telzer EH, Burnell K. Smartphone use during school hours and cognitive control in adolescents. *JAMA Netw Open*. 2026;9(2):e261092.
doi:10.1001/jamanetworkopen.2026.1092

eFigure. Screen Shot

This supplemental material has been provided by the authors to give readers additional information about their work.

© 2026 Telzer EH et al. *JAMA Network Open*.

eFigure. Screen Shot

As shown in the image below (image from the Apple website), adolescents can take a screenshot of their screen time. It depicts the total time spent on their device the prior day (e.g., 2h45m) as well as a graphic of screentime per hour of the day. We created a coding system to extract the hourly data for each hour. Research assistants extracted the hourly data for each hour for each day for each participant. To confirm reliability, we correlated the hourly extracted screentime (summed across the 24 hour period each day) and the screentime metric for the same 24h period provided by apple (e.g., 5h 30m). These values were correlated *r*=1.00, representing perfect reliability, confirming this method provided a reliable metric of hourly screen use.

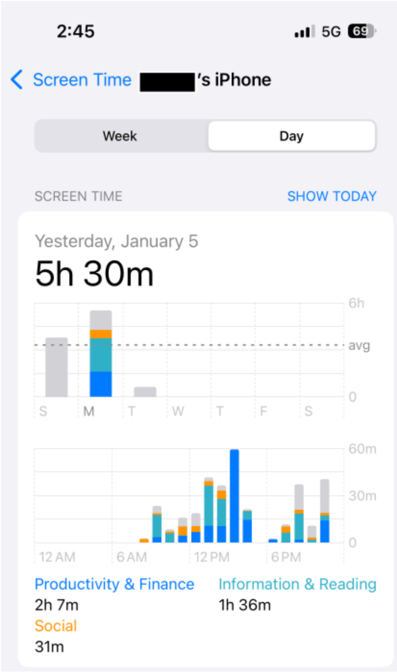

© 2026 Telzer EH et al. *JAMA Network Open*.
