# Supplementary material for: Smartphone Use During School Hours and Association With Cognitive Control in Youths Aged 11 to 18 Years
Source: JAMA Netw Open. 2026 Mar 9;9(3):e261092. doi: 10.1001/jamanetworkopen.2026.1092 (PMC12973097; doi:10.1001/jamanetworkopen.2026.1092)
Supplement: Supplement 2. — Data Sharing Statement [file jamanetwopen-e261092-s002.pdf]

Data Sharing Statement

Telzer. Smartphone Use During School Hours and Association With Cognitive Control in Youths Aged 11 to 18 Years. *JAMA Netw Open*. Published March 09, 2026. doi:10.1001/jamanetworkopen.2026.1092

Data
Data available: No

Additional Information
Explanation for why data not available: Data can be made available upon request
